# Impact of targeted neck and shoulder muscle training on muscular tension in ear surgeons

**DOI:** 10.1007/s00405-025-09596-2

**Published:** 2025-08-20

**Authors:** Antonia Lakomek, Sophia Minhorst, Martin Schulze, Theda Eichler, Moritz Meyer, Benedikt Höing, Kazim Shiraliyev, Stephan Lang, Diana Arweiler-Harbeck

**Affiliations:** 1 Department of Otorhinolaryngology, Head and Neck Surgery, University Hospital Essen, Hufelandstraße 55, 45147 Essen, Germany; 2 Department of Physiotherapy, University Hospital Essen, Hufelandstraße 55, 45147 Essen, Germany

**Keywords:** Ear surgery, Muscle training in ear surgeons

## Abstract

**Purpose:**

Ear surgeons are frequently exposed to prolonged static loads on the neck and shoulders during surgical procedures using a microscope, which can lead to muscular tension and consequently to chronic discomfort and complaints. This study investigates whether targeted training of the neck and shoulder girdle muscles can reduce muscle tension in ear surgeons during and after surgeries.

**Methods:**

For this purpose, four ear surgeons participated in the study, completing an application-based (app) and app-monitored training program for the neck and shoulder muscles over a period of eight weeks. Muscle tension in the trapezius muscle (M. trapezius) and the neck muscles was measured using electromyography during a cochlea implant surgery. Muscle tension was compared before and after the start of the training program. Additionally, the surgeons completed a questionnaire regarding physical activity, stress levels, and other relevant factors.

**Results:**

Results from 100 surgeries show a significant reduction by 20% in trapezius muscle tension due to the training program while performing surgery (*p* = 0.05). Moderate activity on the previous day could reduce muscle tension in the trapezius during surgery by 31% (*p* = 0.01), high activity on the previous day by 20% (*p* = 0.40). All surgeons stated that they had subjectively benefited from the training and that they would voluntarily continue performing the exercises beyond this study.

**Conclusion:**

Targeted training and physical activity on the previous day can reduce muscle tension during surgeries. This highlights the relevance of training programs and moderate activity in reducing strain in daily surgical routines.

## Introduction

Work-related musculoskeletal disorders (WRMD), internationally recognized as such, are an increasing issue in surgery, affecting approximately 60–90% of surgeons [1, 2]. Particularly in otorhinolaryngology (ENT), where long hours are spent at the microscope and twisted body postures during endoscopic surgeries are common, a high prevalence of WRMD is reported in literature. In a large Canadian study by Bolduc-Bégin et al., a survey conducted with ENT specialists found that 97% of participants had experienced musculoskeletal complaints at some point. 74% reported a worsening of symptoms during work, and 25% experienced a significant deterioration of symptoms after surgery. 15% of respondents indicated they had to take sick leave due to WRMD [3].

Boyle et al. (2021) also demonstrated the relevance of WRMD among ENT specialists in a large-scale study, where the prevalence was found to be 75.5% [4]. The literature particularly highlights the neck and shoulder muscles as the most affected areas [3, 4].

Research shows a positive correlation between head flexion of more than 20°, which is typical during microscopy in ENT, and neck pain [5]. Overall, literature highlights the problem of WRMD in the daily work of ENT specialists. While some studies have investigated ergonomic improvement in the operating room - such as microscope upgrades (Lakomek et al.) and supportive seating (Vijendren et al.) - these primarily focus on passive modifications [6, 7]. In addition to this, in a systematic review by Storey et al., various intervention studies aiming at the prevention of work-related musculoskeletal disorders (WRMD) in ENT physicians were examined. However, most of these studies focused on new clinical equipment, patient positioning, and surgical techniques, rather than targeted training for ENT doctors [8].

Resistance training may reduce muscle tension by improving strength and efficiency, thus lowering sustained muscle activation. Andersen et al. showed that brief daily training increased EMG gaps and reduced neck/shoulder pain in office workers [9].

The objective of the present study is to assess the impact of an eight-week neck and shoulder strengthening training program on intraoperative muscle activity, as measured by EMG, in ear surgeons performing microscope-assisted procedures.

## Materials and methods

This study is a single-center prospective, pre-post observational pilot clinical trial, which investigates a potential exploratory improvement in muscle tension among ear surgeons through targeted training using the example of cochlear implantation surgery. The surgeries were performed using a digital tripod microscope with a conventionally anchored ocular. Cochlear implantation surgeries were performed by four experienced ear surgeons (1 female, 3 male). All surgeons from the clinic conducting the study who perform cochlear implant surgeries were included. None of the surgeons had a diagnosed musculoskeletal disorder. Age ranged from the late 30 s to late 50 s and was evenly distributed.

The electrical activity of the neck and shoulder muscles in surgeons was measured using surface electromyography (EMG) during cochlear implantation procedures. The software Biofeedback myoRESEARCH^®^ (Noraxon USA, Scottsdale/Arizona; MR3-software, version 3.20) was used. Within this system, the tool myoMUSCLE^®^ (EMG recordings and biofeedback) was utilized.

Electrical potentials were derived at skin level using electrodes placed on the upper trapezius muscle and the neck muscles and recorded by the Ultium EMG 8-channel system, with four channels (left/right trapezius descendens, left/right cervical spine extensors) [10]. Electrodes were placed according to SENIAM guidelines on anatomical landmarks to ensure standardized, reproducible data. The myoRESEARCH^®^ system by Noraxon uses a self-adhesive electrode with two integrated electrodes at a fixed distance (approx. 20 mm). This sensor pad is applied directly to the target muscle, and the electrode spacing follows SENIAM recommendations. A cable connects the pad to a small recording unit, which is also attached to the skin. Skin at electrode sites was cleaned, shaved, and lightly abraded to reduce impedance and improve signal quality [11].

The electrical activity of the muscles (right and left) was measured separately. The data were transferred to an external computer and displayed as a function over time (unit of measurement: microvolt).

The resting tension of the described muscle groups was measured in a relaxed sitting position for one minute, both before and after the surgery. During the use of the microscope, the muscle tension was continuously measured.

For all the measurements described above, the average voltage was calculated.

In addition, the duration of microscope use, the side of the surgery, the number of previous surgeries performed by the surgeon, and the patient’s age were recorded.

Before the surgery, the surgeons filled out a questionnaire which, using a numerical analogue scale (0 = very low; 10 = very high), assessed the current stress level, the subjective feeling of muscle tension, and the physical activity on the previous day. After the surgery, the participants were required to indicate the difficulty level of the operation. For the analysis, the responses on the analogue scale were categorized into three levels: low (≤ 3), medium (4–6), and high (≥ 7) [12].

After a minimum of five observational measurements during a cochlear implantation surgery for each surgeon, the training period started. In this phase, each surgeon was trained and guided in real-time by a physiotherapist on how to perform the training program. There was also determined, with how much weight the training should be conducted, adjusted to the individual training condition. This was followed by the home training phase, which was managed via the wibbi^®^ app [13]. The app listed and demonstrated each exercise. Surgeons were asked to document all training sessions using this app. Two to three sessions per week of 25 to 30 min each were recommended.

The choice of exercises was based on a systematic review and meta-analysis dealing with interventions for neck pain, which leads to an evidence-based neck training protocol [14]. The cited studies reported a training effect after 8–10 weeks, so a minimum training period of eight weeks was used in the present study. Short vacation periods were included within this period. During this time training was continued, although it was not documented.

After the eight-week training phase, the “post-training”- measurements started. During this period, the surgeons continued training in their own interest, as they subjectively felt very positive about the training.

For analysis, the measurements taken before, during, and after the training were evaluated depending on these three phases.

### Exercise types

During each training session, participants performed the exercises as follows: three exercises were selected from five available exercises for each session. The choice of exercises was varied from session to session. All exercises were initially instructed by a physiotherapist. Participants were instructed to distribute the selections of exercises in a balanced manner across the training days to ensure comparability. The training was performed at high intensity, using 70–80% of the one-repetition maximum (eight to twelve repetitions until fatigue).

**Fig. 1:**
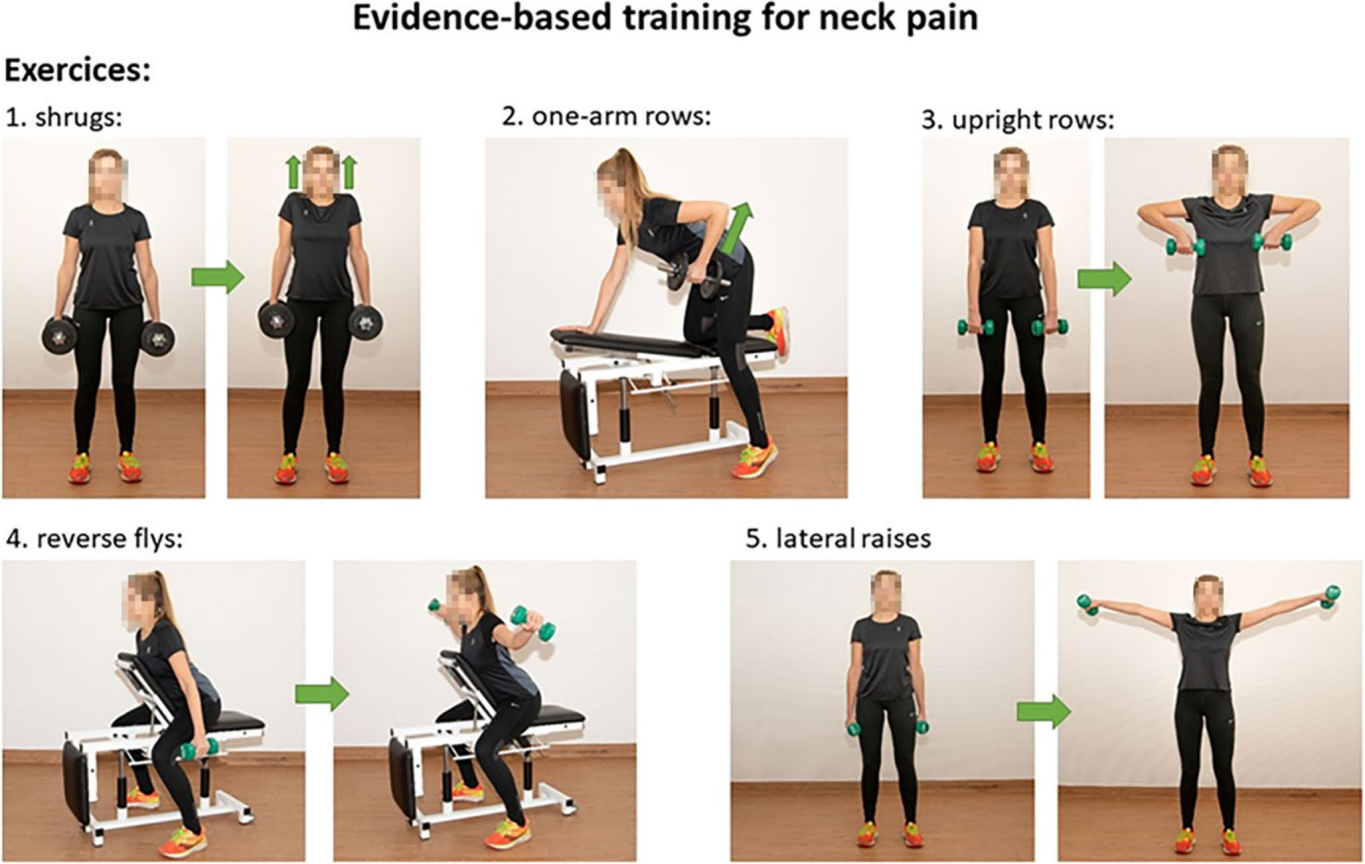
Types of exercises: The figure shows the performance of the exercises from the training program

The following five different neck and shoulder exercises were performed: shoulder shrugs, one-arm rows, upright rows, reverse flys and lateral raises (Fig. 1).

### Questionnaire

After the study, all surgeons were given a questionnaire. This questionnaire assessed changes in subjective muscular tension and overall well-being following training. It included nine questions, utilizing a numeric analogue scale (VAS) from 0 to 10 to measure participants’ perception of improvement in various aspects. Following was surveyed: change in overall well-being, level of physical activity in recent weeks, frequency of headaches experienced in the past, reduction in headache days per month, frequency of back pain experienced in the past, reduction in back pain days per month, perceived improvement in posture while using a microscope, likelihood of continuing training exercises voluntarily beyond the project.

The questionnaire aims to evaluate the subjective benefits of training in reducing muscular discomfort, enhancing posture, and promoting long-term adherence to therapeutic exercises.

### Study specifics

This study uses baseline data from the previously published study “Muscular tension in ear surgeons during cochlear implantations: does a new microscope improve musculoskeletal complaints?” comparing muscular tension in surgeons [6]. However, the present study does not examine the difference between two microscopes but rather whether an intervention in the form of muscular training shows potential for improvement. 33 measurements from only two surgeons were taken from the already published manuscript as pre-measurements for the analyses of the present study (paragraph one of the results of [6]). In total, 100 measurements were included in this study. The use of the 33 measurements was done to enhance statistical power. The actual relevant measurements, that is, those during and after the training phase, are all new and have never been published before.

Since it is a separate article, the results of the study are to be considered hypothesis-generating and exploratory.

### Statistical analyses

For statistical analysis SciPy (Virtanen 2020) and for visualising the data Seaborn (Waskom 2021) was used. T-tests were performed to calculate the significance for independent samples. Measurement values were given as the average muscle tension in microvolt. *n* represents the number of surgeries included in the analyses.

## Results

**Fig. 2:**
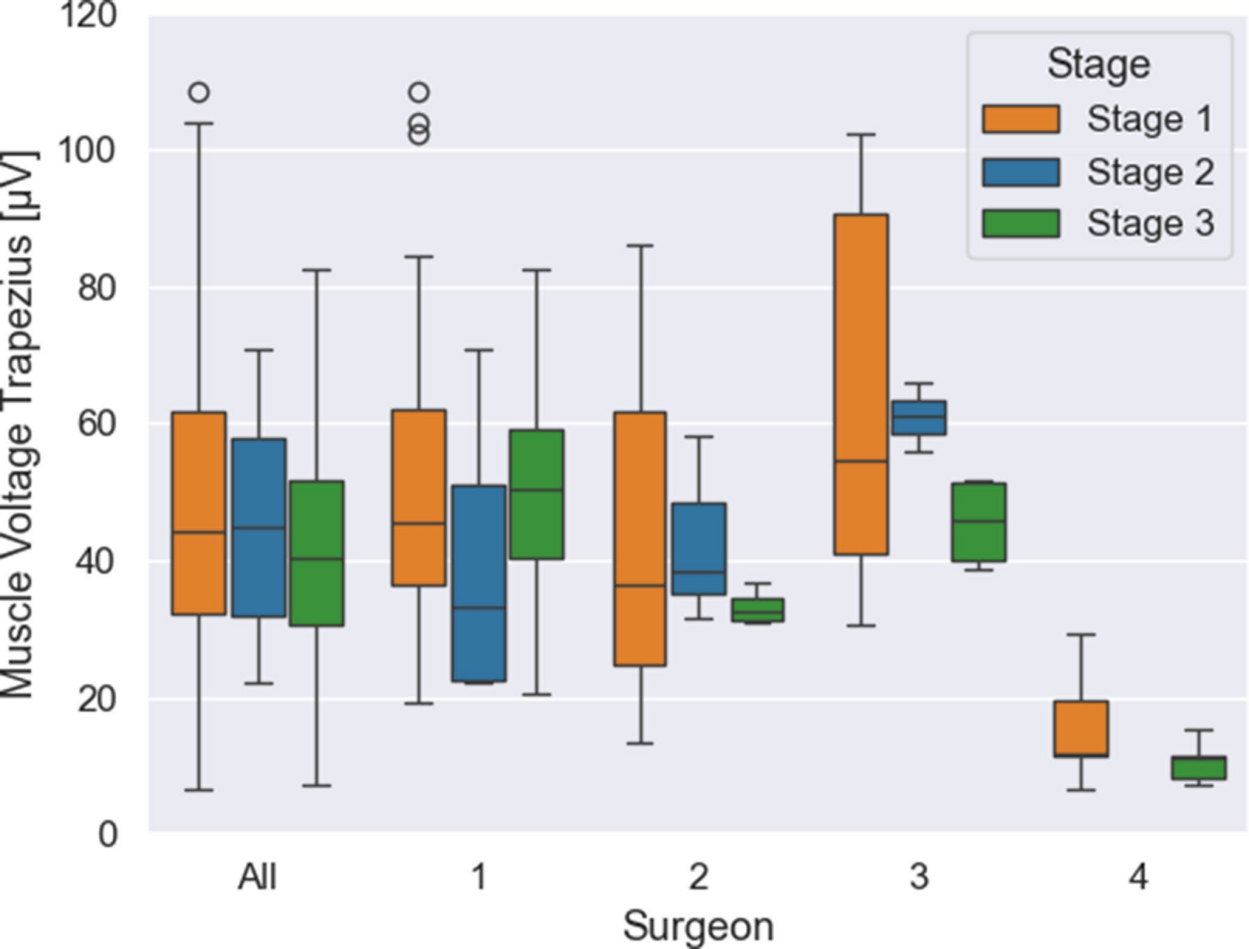
Tension of the trapezius muscle during surgery as a function of training phases: The box plot shows the muscle tension of the trapezius muscle depending on the three training phases, divided into the totality of surgeons and the individual surgeons

A total of 100 cochlear implantations using a conventional digital microscope were included in the study. Averaged across all surgeons, a significant reduction by 20% in trapezius muscle tension was observed from phase one to phase three (stage 1: 48.53 µV; *n* = 65 vs. stage 2: 45.01 µV; *n* = 10 vs. stage 3: 38.82 µV; *n* = 25; *p* = 0.05), (Fig. 2). Regarding the individual surgeons, the tension of the trapezius muscle decreased continuously considered as a trend from phase one to phase three in three out of four cases (surgeon 2: stage 1: 43.71 µV; *n* = 9 vs. stage 2: 42.79 µV; *n* = 3 vs. stage 3: 33.24 µV; *n* = 4; *p* = 0.26; surgeon 3: stage 1: 64.41 µV, *n* = 7 vs. stage 2: 60.97 µV, *n* = 2 vs. stage 3: 45.54 µV, *n* = 4; *p* = 0.15; surgeon 4: stage 1: 15.76 µV, *n* = 5 vs. stage 3: 10.59 µV, *n* = 5; *p* = 0.28), (Fig. 2). In one out of four cases, a reduction in muscle tension was observed particularly from phase one to phase two, while the reduction from phase one to phase three was minimal (surgeon 1: stage 1: 50.72 µV, *n* = 44 vs. stage 2: 39.95 µV, *n* = 5 vs. stage 3: 50.20 µV, *n* = 12; *p* = 0.93), (Fig. 2). No statistical significance could be demonstrated here.

**Fig. 3:**
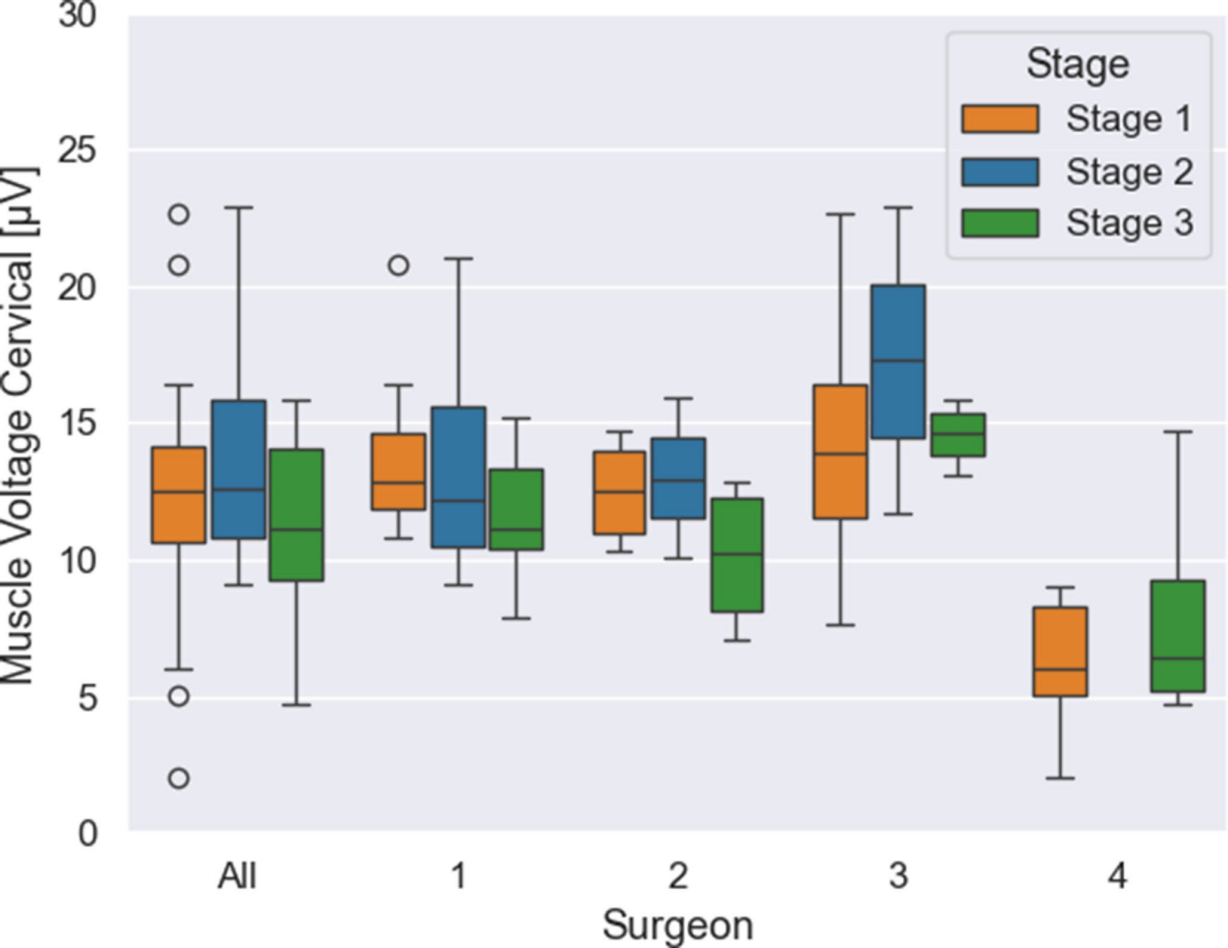
Tension of the cervical muscle during surgery as a function of training phases: The box plot shows the muscle tension of the cervical muscle depending on the three training phases, divided into the totality of surgeons and the individual surgeons

When examining the measurements of the cervical muscles, an overall reduction of tension (considered as a trend) from phase one to phase three by 9% was observed across all surgeons, with a minimal increase in tension in phase two (stage 1: 12.27 µV, *n* = 28 vs. stage 2: 14.19 µV, *n* = 10 vs. stage 3: 11.20 µV, *n* = 25; *p* = 0.31), (Fig. 3).

Concerning the individual surgeons, two out of four show a non-statistically significant reduction from phase one to phase three, with surgeon 1 showing a statistically significant difference (surgeon 1: stage 1: 13.74 µV, *n* = 13 vs. stage 3: 11.75, *n* = 12, *p* = 0.05; surgeon 2: stage 1: 12.50 µV, *n* = 5 vs. stage 3: 10.12 µV, *n* = 4, *p* = 0.20). In contrast, for the remaining two surgeons, no substantial reduction in cervical muscle activity from stage one to stage three was observed (surgeon 3: stage 1: 14.43 µV, *n* = 5 vs. stage 3: 14.55 µV, *n* = 4, *p* = 0.97; surgeon 4: stage 1: 6.08 µV, *n* = 5 vs. stage 3: 8.08 µV, *n* = 5, *p* = 0.40). Surgeon number four did not perform cochlea implants during stage two.

**Fig. 4:**
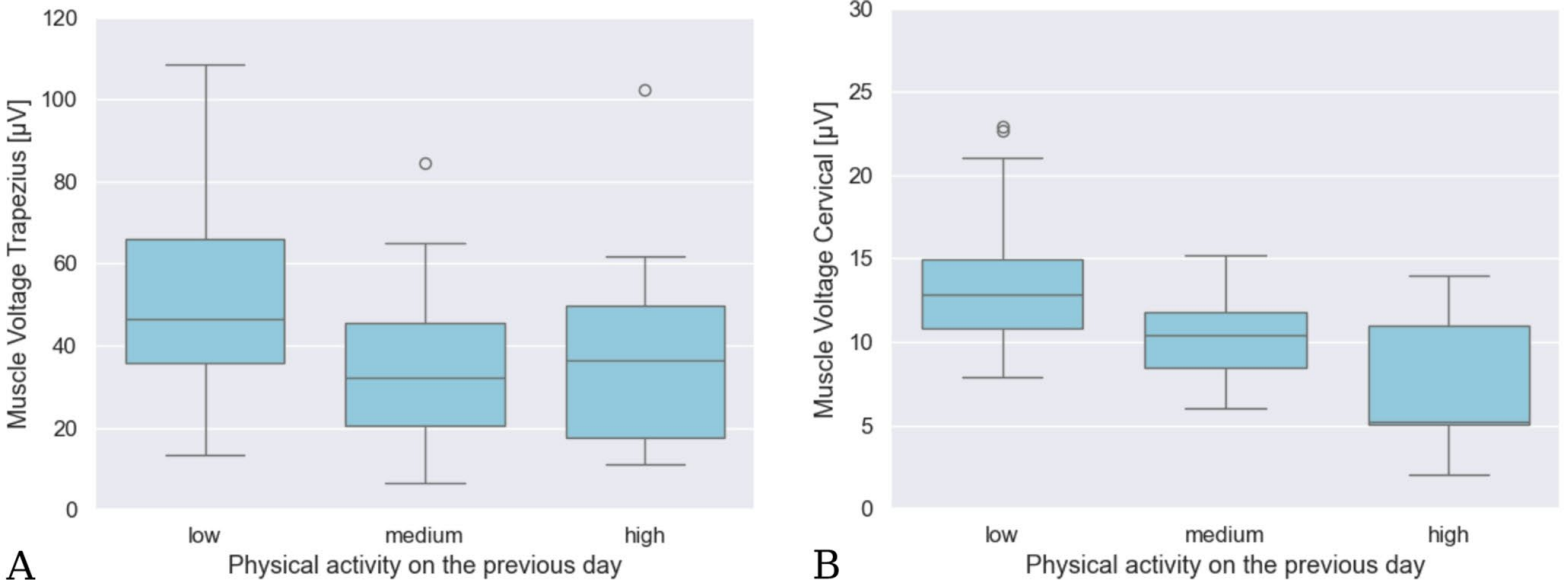
Tension of the trapezius (**A**) and the cervical muscle (**B**) during surgery in relation to the physical activity on the previous day: The figure shows in part A the average muscle tension of the trapezius muscle and in part B that of the cervical muscles, depending on the physical activity level of the previous day, which is categorized into low, medium, and high

Regarding physical activity on the previous day across all phases combined, measurements demonstrated that moderate to high physical activity on the preceding day was associated with significant reduction of muscle tension during surgery in both the trapezius muscle and the cervical muscles. In detail, muscle tension in the trapezius during surgery was reduced by 31% (*p* = 0.01) following moderate activity on the previous day and by 20% (*p* = 0.40) following high activity. Regarding the cervical muscles, moderate activity on the previous day was associated with a reduction of 23% (*p* = 0.01) in muscle tension, while high activity resulted in a 44% (*p* = 0.05) reduction (Fig. 4).

**Fig. 5:**
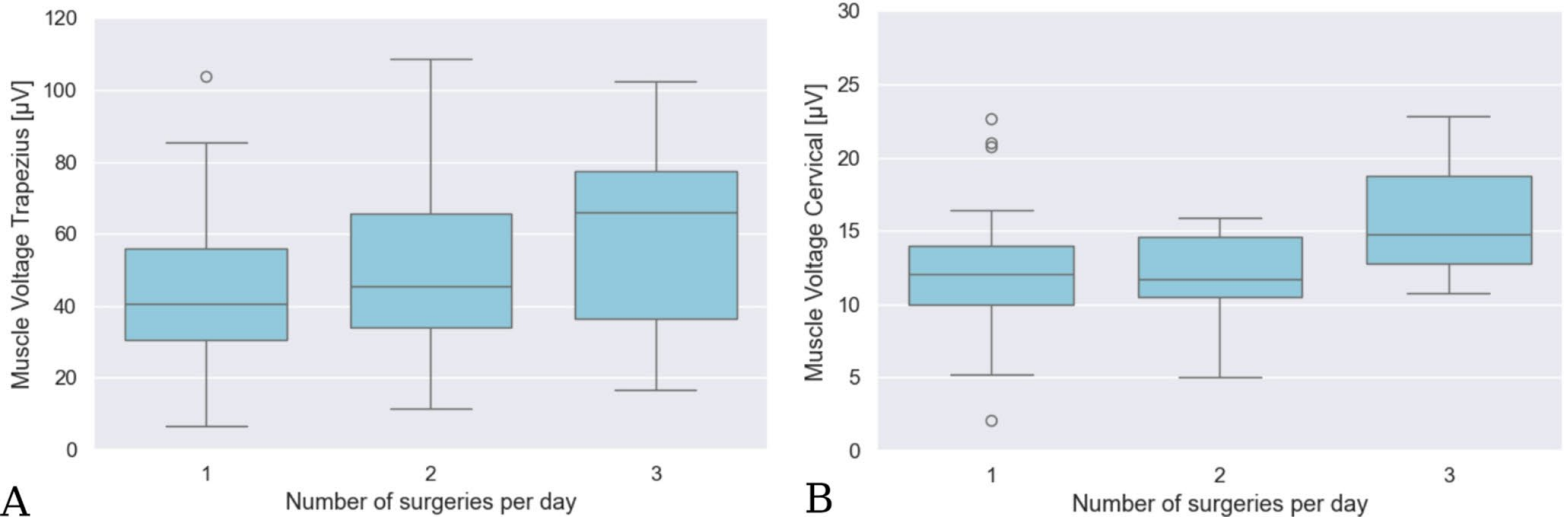
Tension of the trapezius (**A**) and the cervical muscle (**B**) during surgery in relation to the number of surgeries per day: The figure shows in part A the average muscle tension of the trapezius muscle and in part B that of the cervical muscles, depending on the number of surgeries already performed on the day (on the left when the measured surgery was the first, in the middle when the measured surgery was the second, and on the right when the measured surgery was the third for the surgeon on that day)

With respect to the number of surgeries performed on the same day before the measured surgery, there was a non-statistically significant increase in muscle tension in both the trapezius muscle and the cervical muscles when the measured cochlear implantation was the surgeon’s third operation on this day (trapezius muscle: first surgery 43.21 µV, *n* = 55 vs. second surgery: 50.92, *n* = 31 vs. third surgery 59.82 µV, *n* = 5; p-value first vs. third = 0.34; cervical muscles: first surgery 12.10 µV, *n* = 37 vs. second surgery 11.90 µV, *n* = 15 vs. third surgery: 16.12 µV, *n* = 3; p-value first vs. third = 0.38) (Fig. 5).

A high subjective stress level of the surgeons was linked to a lower baseline muscle tension during the performed operation in both measured muscle groups compared to a low stress level (trapezius muscle: low: 49.55 µV, *n* = 68 vs. high: 30.79 µV, *n* = 7, *p* = 0.21; cervical muscles: low:12.49 µV, *n* = 40 vs. high: 7.20 µV, *n* = 6, *p* = 0.04).

Since the beginning of the training, a total of 64 training sessions were recorded in the app across all surgeons. No recordings were made during vacation periods of approximately around six weeks of all surgeons during this time. The intended frequency was 2–3 sessions per week. Three surgeons trained on average about twice per week and one surgeon trained on average about two to three times per week (excluding vacation time). Across all training sessions, the selection of performed exercises was evenly distributed for each surgeon. Only the third surgeon did not perform any lateral raises.

In the final questionnaire, which all four surgeons completed, a significant self-reported improvement in muscle tension and overall well-being was shown.

Concerning the perceived reduction in tension, the surgeons reported scores of at least 6/10, with a mean value of 7/10 on the numeric analogue scale. The enhancement in self-reported well-being was indicated by scores of at least 5/10, averaging 7/10. Two out of the four surgeons regularly suffered from headaches prior to the study. For these two, the headaches were self-reported reduced through the training (numeric analogue scale: seven). All four surgeons rated at least a five for the question about regular back pain, with an average of seven. Three out of four surgeons were able to subjectively perceive a reduction of days they suffered from back pain through the training (numeric analogue scale: average eight). All surgeons reported that their posture at the microscope had improved through the exercises (average: 8/10). All surgeons indicated that they would voluntarily continue the exercises, even after the study finished (min. 5/10, average: 8/10).

## Discussion

The present study demonstrates the relevance and necessity of preventive physical exercises s for surgeons to reduce muscular strain in daily professional practice.

Many studies have shown that WRMD are a major issue in surgical practice [1–4], but only a few studies have demonstrated preventive measures to minimize physical strain [6, 7].

In this study it could be shown that preventive targeted training of the shoulder and neck muscles is an opportunity to significantly reduce muscular strain of the musculus trapezius by 20% and of the cervical muscles by 9% during microscope-assisted surgery. The group analysis shows statistical significance, while the individual analyses only showed a trend. This discrepancy may be due to the statistical power, which is considerably higher in the group analysis.

Interestingly, a reduction in phase three could not be demonstrated for the trapezius muscle in only one surgeon. However, this surgeon showed a significant reduction in muscle tension in the cervical region. This surgeon performed all exercises evenly distributed and did not have a concentration of neck or shoulder muscle exercises, so that apparently no influence was caused by this. This may imply that, for example, the cervical strain was particularly high before training and was reduced through the intervention. Similarly, no measurable change in cervical muscle tension was detected in two other surgeons. However, both showed a significant reduction in muscle tension in the trapezius muscle. Overall, this suggests that certain muscle groups are individually subjected to higher strain than others. This could further indicate that preventive training should be adapted based on individual strain conditions. This is already a well-researched topic in elite sports [15]. Additionally, factors such as sex, age, and weekly surgical hours may also influence the training effects observed. For instance, differences in muscle activation or fatigue patterns could be related to physiological differences or workload demands. However, given the sample size of four participants, these considerations should be interpreted with caution. Further research with a larger and more diverse cohort is needed to investigate these potential influences systematically. When examining the cervical muscle group, there was a slight, short-term increase in muscle tension at the beginning of the training phase. This is likely due to a temporary adaptation of the muscles in response to the new demands [16]. According to many general studies, physical activity can reduce muscle tension and pain [17]. The results presented here demonstrate this relevance in the context of reducing muscular strain during surgical tasks. This relatively simple method of regularly integrating physical activity into the workday or leisure time should be given greater emphasis in the prevention of work-related ailments in surgeons.

As expected, the muscular strain of the surgeons increased after having performed two surgeries. This demonstrates the physical strain caused by prolonged surgical activities.

Surprisingly, muscle tension during surgery was lower at a preoperatively assessed high stress level than at a low stress level. This inverse observation could possibly be explained by the fact, that in the presence of pronounced external stress factors, there is a conscious effort made to relax and focus on the upcoming surgery. Consider psychological mechanisms such as attentional focus, stress-induced coping strategies, or autonomic regulation to explain this counterintuitive result. According to Lazarus and Folkman’s transactional model of stress and coping, individuals’ appraisal and coping efforts can modulate physiological stress responses [18], potentially leading to reduced muscle tension despite elevated perceived stress. Moreover, autonomic nervous system regulation, including parasympathetic activation, can facilitate muscle relaxation even under psychological stress (Thayer & Lane) [19]. When interpreting the results, the sample size regarding the topic of stress must especially be considered.

Although filling out a questionnaire is often subject to personal feelings, the final questionnaire was able to independently demonstrate a positive effect of the training for each surgeon.

For all surgeons, the training subjectively resulted in better posture at the microscope, a reduction in the feeling of tension and a reduction in back pain.

It is well known that tension in the shoulder and neck muscles can lead to headaches [20]. Therefore, it is an interesting observation that the two surgeons who had suffered from regular headaches before the study experienced a reduction in these symptoms through the training.

One of the most important statements from the questionnaire was that the participants would like to continue the training even beyond the study. This indicates that the exercises are perceived as a subjective benefit rather than an additional burden. Concerning further adherence to such measures, also outside of studies, this is an important finding.

It can be stated that targeted muscle training and physical activity in everyday life are important and significant preventive factors for muscle tension during surgical activities. The exercises performed are low in time intensity, easy to implement, cost-effective and have a significant effect in our study. For the reduction of muscle tension shown, no special equipment in the operating room is needed, unlike to other studies that have dealt with this topic [6, 7]. However, despite the relatively low time commitment, there needs to be space in everyday life for physical activity and training. In many companies this is already a common practice. In hospitals in Germany, however, this is a rare occurrence. This highlights the relevance that hospitals, like businesses, will need to create opportunities, such as fitness rooms or discounted gym memberships, for their employees to maintain physical health preventively. Generating hypotheses, this could prevent fewer sick leaves and lead to a prolonged preservation of the workforce’s productivity.

### Strengths and limitations

The study is subject to the typical limitations of a prospective single-center study with a small study population. Although a total of 100 measurements during cochlear implantations, with around 35 measurements from the training period is relatively high, the limited number restricts statements about statistical significance. The relatively higher number of pre-measurements was ensured by incorporating data from another study on this topic. Additionally, we included four highly experienced surgeons and despite measuring 100 surgeries, intra-subject variability and phase imbalances limit the robustness of the results. The results of this study should be considered as hypothesis-generating, and further studies with more participants should follow. The study examines short-term success using the presented methods. Long-term success cannot be derived from this study. Future research should incorporate long-term follow-up and objective functional outcomes, such as work absenteeism and surgical performance metrics, to evaluate the durability of the observed effects more comprehensively.

All information from the questionnaires should be considered subjective.

## Conclusion

Focused training and physical activity the day before surgery could be associated with reduced muscle tension during surgical procedures. This underscores the importance of training programs and moderate activity in alleviating strain during daily surgical routines. An additional approach to improving muscular strain is direct biofeedback during surgery. This is currently being investigated in an ongoing study. While the results are promising, the sample size, lack of a control group, and single-center design limit their generalizability.

## Declarations

This study was performed in line with the principles of the Declaration of Helsinki.
